# Medicines and society: systemic change needed to address overprescribing

**DOI:** 10.3399/BJGP.2025.0050

**Published:** 2025-06-27

**Authors:** Caroline McCarthy

**Affiliations:** Department of General Practice, Royal College of Surgeons in Ireland (RCSI) University of Medicine and Health Sciences, Dublin 2, Ireland

Overprescribing occurs when medicines are prescribed that are ineffective, have an unfavourable risk–benefit ratio, do not align with patient preferences, or where a better nonmedical alternative exists.^1^ Estimates suggest that up to 10% of medications are overprescribed.^1^ Interventions to address overprescribing (and its many synonyms, such as potentially inappropriate prescribing and low-value prescribing) have typically focused on changing healthcare professionals’ prescribing behaviours. While these efforts are important and effective, this article argues that systemic changes are also needed to successfully address rising levels of overprescribing.

Increased consumerism, often supported by pharmaceutical funding and patient support groups, has contributed to a widespread belief that many of life’s challenges can be treated or addressed medically. This perpetuates narratives that medicalise normal physiological processes or life experiences, and that a pharmacological solution exists for every symptom, while simultaneously distracting from non-pharmacological management approaches and preventing disease by addressing the social determinates of health. This perspective is particularly apparent in the management of conditions that are diagnosed based on subjective symptoms. For example, emotional distress may be labelled as clinical depression, with roughly 20% of the adult population in England prescribed selective serotonin reuptake inhibitors in 2022.^2^ Functional dyspepsia, gastro-oesophageal reflux disease, gastritis, and peptic ulcer disease are on a continuum of severity but only a minority of patients presenting with related symptoms are at the extreme end of the spectrum. In addition, many of these symptoms can be addressed with simple lifestyle and dietary modifications; however, the prescription of proton pump inhibitors has increased by over 50% in the past 9 years despite guidance designed to address their overprescription.^3^ These statistics are examples of a trend towards medicalising conditions that may be better addressed through non-medical interventions. Addressing the societal and medical risks associated with this overmedicalisation and overtreatment has emerged as a priority and has been tackled by various international initiatives within the research and clinical communities, including campaigns such as ‘Choosing Wisely’ and the *British Medical Journal*’s ‘Too Much Medicine’.^4,5^

A cultural and educational shift that includes promoting public discourse and scrutiny of medical practices, addressing conflicts of interests in medicine, and implementing public health policies to address broader determinants of health are all essential approaches to tackle overprescribing.

## Medical education

### Numerical and probabilistic reasoning

Research assessing the numeracy skills of foundation registrar doctors in the UK highlighted that a significant proportion of graduates struggled to identify the better treatment option when data were presented as relative risk reduction, absolute risk reduction, and number needed to treat.^6^ Accurate interpretation and effective communication of risk is essential to support patients in making informed, preference-sensitive decisions. Strong numerical reasoning skills are critical for facilitating shared decision-making and ensuring patient-centred care (Box 1).^7–9^ While this approach helps patients gain a sense of the likely benefit, supporting informed decision-making, it is important to acknowledge that it remains a simplification. Randomised controlled trial data typically present average results, meaning estimates reflect an average effect, whereas individual responses may vary, with some patients benefiting more than others. However, this approach is important in addressing overprescribing as there is evidence that, when the benefits of preventive medicines are presented in absolute, rather than relative terms, patients are often less likely to accept treatment.^10,11^

Box 1 **Therapeutic decision-making scenarios highlighting the importance of numerical reasoning**

1. Cholesterol levels with varying CVD risk profiles:^7^
  - Patient A: 53-year-old woman from an affluent area, with a cholesterol/HDL ratio of 7, systolic BP of 130 mmHg, and a BMI of 31.
  - Patient B: 55-year-old woman from an area of deprivation, who is a heavy smoker, has a family history of CVD, and personal history of hypertension, with a cholesterol/HDL ratio of 4, systolic BP of 130 mmHg, and a BMI of 25.
  - Clinical question: Do I need a statin?
  - Numerical insight: Patient A’s risk of CVD is reduced by 1% over 10 years whereas patient B’s risk is reduced by 10%. One hundred patient As would have to be treated for 10 years to prevent one bad outcome, compared with 10 patient Bs.^8^
2. Osteoporosis with varying fracture risk profiles:
  - Patient A: 70-year-old woman with a BMD T-score of −2.5, no previous fractures, no family history of osteoporosis, and generally healthy lifestyle.
  - Patient B: 65-year-old man with a BMD T-score of −2.0, history of smoking, previous wrist fracture, and long-term glucocorticoid use.
  - Clinical question: Do I need a bisphosphonate?
  - Numerical insight: For patient A the ARR of hip fracture with bisphosphonate use is 1.2% over 10 years; for patient B the ARR is 2.8%. Eighty-three patient As compared with 36 patient Bs would have to be treated for at least 5 years to prevent one hip fracture.
3. Breast cancer treatment with intolerable side effects from tamoxifen:
  - Patient: Woman with node-negative oestrogen receptor-positive breast cancer experiencing severe side effects from tamoxifen.
  - Clinical question: What is the risk of stopping this medicine?
  - Numerical insight: ARI of breast cancer recurrence over 5 years upon stopping tamoxifen is 5.6%. For every 18 similar women who stop their tamoxifen one would have a recurrence of her breast cancer that she would otherwise have avoided.^9^

ARI = absolute risk increase. ARR = absolute risk reduction. BMD = bone mineral density. BMI = body mass index. BP = blood pressure. CVD = cardiovascular disease. HDL = high-density lipoprotein.

### Social determinants of health

Social determinants of health (SDH) are increasingly recognised for their substantial impact on health outcomes, with clinical care contributing only about 10%–20% to these outcomes.^12^ Recent shifts towards integrating SDH into medical education highlight the essential role these factors play in both diagnosing and treating patients. While acute medicine is necessary to help patients with chronic illness and those who are currently unwell or at high risk of illness, understanding the role of SDHs helps identify individuals at heightened risk of morbidity and mortality while acknowledging the limited reach of clinical care alone in improving overall health at both individual and population levels. Using childhood asthma as an example, there is evidence that effective home heating is associated with reduced exacerbations.^13^ Although not overprescribing in the strictest sense as medications are clearly needed to treat exacerbations, but if a long-term, non-medical solution exists and is not implemented, continued treatment in this context constitutes overprescribing. To better equip future doctors to recognise and address SDH, public health placements should be an integral part of both undergraduate and postgraduate medical education.

### Narrative medicine

Doctors are trained to take structured histories, formulate a differential diagnosis, and propose a management plan. This biomedical paradigm is interventionist by nature and may contribute to overprescribing; indeed, the ‘desire to do something‘ has been identified as a barrier to avoiding low-value care.^14^ Ethnographic research has indicated that, during medication reviews with older patients, existential concerns are often aired and witnessed, yet these concerns frequently remain unaddressed because of tension with the biomedical paradigm, which prioritises risk reduction and medical intervention over broader discussions of meaning, ageing, and quality of life.^15^ Cultivating the ability to connect with patients and engage with their experiences, without feeling compelled to ‘fix’ every issue with a prescription, is an essential skill to avoid overprescribing. This approach involves teaching future clinicians to be curious and attentive to the detail of the patient’s story, yet the current emphasis on prevention and risk stratification directly impedes this approach.

## Shifting medical culture

### Limits of modern medicine

There is a broader cultural belief that medicine can provide a solution for all health problems. This is evident in mainstream media where the potential benefits of new drugs are often sensationalised. At the individual consultation level, accepting diagnostic uncertainty and the lack of effective treatments when they are unavailable is essential for shared decision-making and avoiding overprescribing. Understanding patients’ expectations about treatments is important as it enhances shared decision-making and provides an opportunity to clarify misunderstandings or discuss misinformation. Actively listening to patients, acknowledging their symptoms, and validating their experiences, even when effective treatments are limited, can be therapeutic.^16^ However, efforts to prevent overtreatment and overprescribing within the consultation are shaped by this broader cultural influence, and, without shifting this overarching narrative, changes at the individual consultation level may have limited impact.

### Interdisciplinary approaches

While GPs are well placed to offer a holistic view of patient care, their primary training and perspective remain largely biomedical. Empowering other allied healthcare professionals (AHPs), who may bring different perspectives and expertise, could help counterbalance this. For example, direct-access physiotherapy may be associated with less medication use and lower overall healthcare costs,^17^ and the implementation of clinical pharmacists in primary care in the UK has been successful and has potentially improved prescribing quality.^18^ However, while interdisciplinary approaches have potential to address overprescribing, there is also a risk that narrowly focused assessments by AHPs within rigid, protocol-driven frameworks may inadvertently contribute to overprescribing.

### Participatory approaches to address overprescribing

Given the broader systemic factors that influence overprescribing, participatory interdisciplinary collaboration will be key to effectively addressing this problem. For example, a sociological perspective may frame overprescribing as a consequence of the organisational, financial, and cultural attributes of the system, not just individual interactions. The perspective of a policymaker may include practical considerations of how changes can be implemented system wide. Participatory action research emphasises local knowledge, shared ownership, and active participation, and this approach has had success in improving antimicrobial stewardship.^19^ In some cases, rigorous effectiveness research may be unnecessary or even wasteful, particularly when the value of an intervention is self-evident. For example, while there is research validating the effectiveness of empathy and interventions to address loneliness, the inherent worth of these approaches is something we intuitively understand as human beings.^20,21^ Access to community resources for patients who are isolated or listening to and empathising with patients who are experiencing chronic pain are clearly important regardless of their impact on measurable outcomes, and these approaches may well help address overprescribing for conditions such as depression or chronic pain. Focusing on implementation and participatory research might be more appropriate to ensure these well-understood benefits are effectively integrated into patient care.

## Societal discourse and healthcare consumption

### Preventive medicine

Societal attitudes towards health and medicine influence healthcare policies and individual behaviours. At the healthcare system level, the current focus on preventive medicine has significant resource implications for primary care to the extent that there has been a recent call to consider the clinician time needed to implement such recommendations.^22^ In addition to the resource implications, *ad hoc* preventive care provided through general practice may promote unnecessary treatment, potential harm, and inequity, with prostate cancer screening serving as a prominent example.^23^ Those who are already well-informed and resource-rich benefit most, thereby widening the health gap between them and marginalised populations who in turn are most affected by shortages of appointments for chronic disease management and acute medical needs. This focus not only has resource and equity implications, but it also potentially turns large numbers of people into patients. There are plenty of examples of evidence-based and effective preventive care. However, the pendulum has swung so far now that the adage ‘prevention is best’ has become an unquestioned truth. In effect, this narrative encourages healthy people to seek medical attention. This coupled with guideline-driven care and a litigation and blame-based culture means GPs are less likely to hold risk and more likely to intervene. The positive predictive value of diagnoses in this context will naturally be lower and the likelihood of resultant overtreatment higher.

### The role of the pharmaceutical industry

The prevailing public narrative that suggests a pharmaceutical solution exists for every ailment may reflect the extensive influence of pharmaceutical industry marketing. This is playing out currently with controversy surrounding the marketing of weight loss medications. Novo Nordisk, the primary producer of these medicines, has recently been sanctioned by the UK pharmaceutical industry regulator over the way it has promoted these medicines including through mainstream media.^24^ The effects of these campaigns shift public discourse towards access to these drugs on state-funded medicines schemes rather than public health measures to address obesity. Like obesity, chronic pain is a common and complex phenomenon that has been heavily influenced by the pharmaceutical industry who promoted its assessment and treatment through a primarily biomedical lens. This approach played a significant role in the US opioid crisis, resulting in widespread harm due to the excessive use of opioid analgesia.^25^ These examples highlight the need for effective regulation of pharmaceutical marketing practices.

### Healthcare system drivers of overprescribing

Beyond the pharmaceutical industry’s influence, the structure of many health systems contributes to overprescribing. In systems with fee-for-service funding models, there is an inherent financial incentive to increase the volume of services, including diagnostic tests and treatments, regardless of their necessity. This can lead to a culture where the default approach is to prescribe or intervene, rather than adopting a wait-and-see approach or focusing on lifestyle changes and preventive measures. In addition, medications that were once the preserve of specialist care are increasingly initiated on the recommendation of secondary care and continued in general practice. This shift risks normalising the use of specialist medicines and may contribute to indication creep as their prescribing becomes more routine in primary care, with examples evident for antipsychotic use.^26^ Recognising and addressing these systemic factors is crucial for developing strategies that aim to reduce overprescribing.

## Call to action

**Figure 1:**
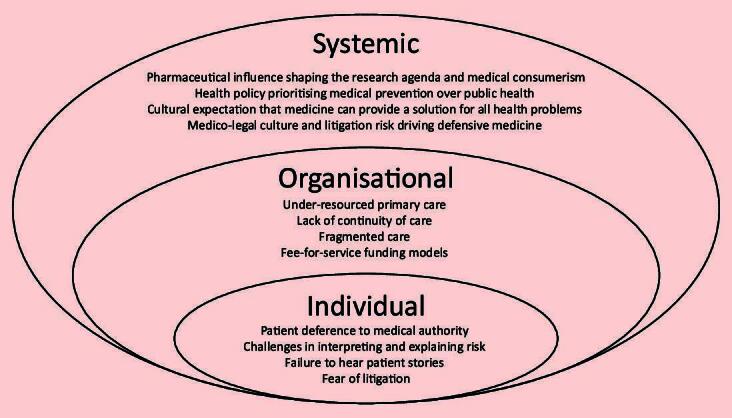
Systemic, organisational, and individual drivers of overprescribing

Upstream systemic factors have a significant impact on overprescribing (Figure 1). Addressing these rather than focusing solely on the downstream factors at the point of care will be necessary to successfully tackle overprescribing. Recently, various organisations and initiatives have advocated for similar approaches. The *National Overprescribing Review Report* in the UK, among its many recommendations, has called for a cultural change and a focus on social prescribing.^1^ In response to this report, the UK College of Medicine and Integrated Health launched its Beyond Pills campaign, advocating for a de-medicalised and community-centred response to emotional distress. More generally, deprescribing networks in both Canada and Australia have advanced the science and practice of deprescribing through research, education, and advocacy. Acknowledging the limits of modern medicine while validating patient experiences is essential to prevent overprescribing. Transforming societal narratives around medicines can create a healthcare environment that reduces overprescribing and enhances the overall quality of care.

**Key messages**

- Overprescribing has important consequences for individuals and healthcare systems.
- Public discourse around the role of medicines influences both patients and prescribers, and can contribute to overprescribing.
- Numerical reasoning and understanding social determinants of health should be integral parts of medical education to support informed, preferencesensitive decisions around medicines use.
- Accepting and acknowledging the limits of modern medicines while validating patients’ experiences and symptoms is crucial to avoid overprescribing.
